# Effects of misinformation diffusion during a pandemic

**DOI:** 10.1007/s41109-020-00327-6

**Published:** 2020-10-31

**Authors:** Lorenzo Prandi, Giuseppe Primiero

**Affiliations:** Department of Philosophy, University of Milan, Milan, Italy

**Keywords:** COVID-19, Misinformation, Misinfodemic, Multi-agent system

## Abstract

The role of misinformation diffusion during a pandemic is crucial. An aspect that requires particular attention in the analysis of misinfodemics is the rationale of the source of false information, in particular how the behavior of agents spreading misinformation through traditional communication outlets and social networks can influence the diffusion of the disease. We studied the process of false information transmission by malicious agents, in the context of a disease pandemic based on data for the COVID-19 emergency in Italy. We model communication of misinformation based on a negative trust relation, supported by findings in the literature that relate the endorsement of conspiracy theories with low trust level towards institutions. We provide an agent-based simulation and consider the effects of a misinfodemic on policies related to lockdown strategies, isolation, protection and distancing measures, and overall negative impact on society during a pandemic. Our analysis shows that there is a clear impact by misinfodemics in aggravating the results of a current pandemic.

## Introduction

The importance of clear and fast communication of policies by governments during a pandemic has been evident on an unprecedented scale during the spread of COVID-19. But the COVID-19 pandemic has also caused an incredible diffusion of conspiracy theories and fake news of any sorts. Misinformation has concerned all facets of the pandemic: well-known are the theories that relate the origin of the virus to its creation in laboratories, and many are the prodigious antidotes proposed to cure or prevent the infection, many of these missing any scientific basis.

In this paper, we are specifically interested in the spread of misinformation resulting from scepticism towards policies and proper behaviour guidance by governments. Assessing the role of misinformation diffusion is crucial for its impact on policies aimed at limiting the circulation of the virus, for example by imposing the use of personal protective equipment (PPE), distancing measures and lockdown strategies. The literature has investigated this problem in a number of ways and through different metodologies: data analysis from a variety of sources, interviews, telephone surveys, online experiments with participants, and also agent-based models (Bridgman et al. 2020; Hameleers et al. 2020; Prati et al. 2011; van der Meer and Jin 2020; Kim et al. 2019). Misinformation of this sort has been well documented: from claims that the virus does not actually exist, to allegations that COVID-19 death rates are inflated, conspiracy theorists have formulated criticisms of social distancing and other protective measures, as well as claims that lockdown and similar measures are entirely unnecessary, see https://www.bbc.com/news/technology-52517797 and Dickson (2020). Another kind of extensively spread type of misinformation concerned the possibility that masks are actually dangerous (Goodman 2020). This kind of news can influence citizens and lead them to act in irresponsible and harming ways for themselves and others. Our aim in this study is to define and quantify such impact.

An aspect that requires particular attention in the analysis of misinfodemics is the rationale of the source of false information, and how the behavior of agents spreading it through traditional communication outlets and social networks can influence the diffusion of the disease, by altering the effects of scientifically proven containment strategies. In Kim et al. (2019), the effect of media on disease spread is approximated relying on real data about disease coverage in the news and a network-based model is offered in which disease is transmitted through local interactions between individuals and the probability of transmission is affected by media coverage. It shows that incorporating media coverage of the outbreak better estimates the disease dynamics. A counterpart of this research, offered in our paper, is an analysis of the effect of misinformation diffusion on the spread of a pandemic through an agent-based model and simulation. Specifically, we studied the process of information transmission in the presence of malicious agents spreading potentially false information by rejecting the content of policies dictated by the authority, and considered their effects on a population roughly divided into three main demographic groups: workers, students and elderly. We investigate these effects on policies related to: lockdown strategies, detection and isolation protocols, protection and distancing measures, and overall negative impact on society. Our analysis shows that there is a clear impact by misinfodemics in aggravating the results of a current pandemic.

The paper is structured as follows. In “A model of misinfodemic by paranoid agents” section we illustrate the design of our model, which includes a hierarchical structure of agents with three demographics and three distinct epistemic attitudes towards information received. In “Simulation of a pandemic with misinformation” section we present our agent-based simulation through an analysis of the algorithmic translation of the model, the parameters used and the properties implemented. In “Results” section we analyze the experimental results of the simulation considering the effects of the misinfoodemic on: the efficiency of safety measures; the efficiency of various lockdown strategies; detection and isolation policies; the overall negative impact of the pandemic on society. In “Conclusions” section we summarize our results and illustrate future direction of research.

## A model of misinfodemic by paranoid agents

We start by modelling our scenario of interest with a logic that accommodates, via formal rules, the behaviour of three types of agents: *paranoids*, *standards* and *sceptics*. We build on the logic $\texttt {SecureND}^{\texttt {sim}}$ (Primiero et al. 2017), which is modelled specifically for simulation purposes, and its negation complete fragment (un)SecureND (Primiero 2016, 2020). The full logic underlying the current study is reported in Prandi (2020): it models a relation of negative trust towards the authority, supported by findings in the literature that relate the endorsement of conspiracy theories with low trust level towards institutions and powerful actors, allegedly being involved in nefarious plots (Abalakina-Paap et al. 1999; Bruder et al. 2013; Douglas et al. 2019; Goertzel 1994; Mashuri and Zaduqisti 2014; Miller et al. 2016; Parsons et al. 1999).

The logic models access control operations of read and write, intended respectively as operations of message receiving and message passing between agents. In this context, information transmission is qualified based on the intentionality of the sender in communicating false information, see Primiero and Kosoloski (2013, p. 254):

- an information transmission that is deemed to convey unintentionally false information is characterised as mistrustful;
- an information transmission that is deemed to convey intentionally false information is characterised as distrustful;
- a receiver that assesses an information transmission as mistrustful or distrustful operates on its content accordingly with operations of accepting or rejecting information.

Hence, operations of message passing are completed by:

- a trust function: a message read by an agent and consistent with the current information she holds, is accepted and passed to the next agent;
- a mistrust_standard function: a message read by a standard agent who receives it from above in the hierarchy and which is inconsistent with the current information she holds is accepted, but requires removing previously held information;
- a distrust_paranoid function: a message read by a paranoid agent who receives it from above in the hierarchy is always rejected;
- a distrust function: a message read by an agent who receives it from below in the hierarchy, and which is inconsistent with the current information she holds is rejected.

These different operations are initiated according to the epistemic characterization of the agents, combined with their position in the hierarchy, i.e an order based on their reputation in the population. Agents higher in the hierarchy are meant to simulate the authorities. For each breed of agents (paranoids, standards and sceptics), a different behavior is modelled. The paranoid agent mimics the behaviour of a conspiracy theorist who does not accept consistent information when it comes from above in the hierarchy: this is a consequence of the low trust levels that she holds towards more reputable agents, suspected of being malicious. In this sense, the paranoid is distrustful of senders higher in the hierarchy, as she attributes them the intention to distribute false information; and she is the source of misinformation, as she might genuinely believe truthful the information she is spreading by negating what she received from the authority. A standard agent is more prone to trust the authority, and she will mistrust information she currently holds when inconsistent with information provided by the authority, i.e. she will be wiling to change her mind. A standard agent might also be näive, and trust a paranoid if this happens to be considered a reputable agent independently of her epistemic characterization. However, a subset of standard agents are sceptic agents: members of this breed are able to recognize paranoids, and will reject information received by them.

**Fig. 1:**
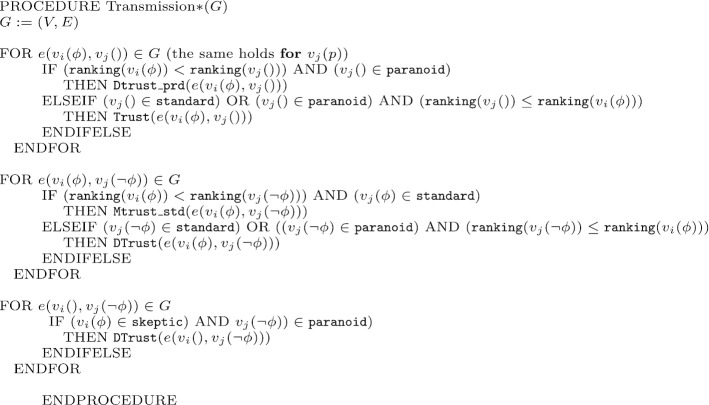
Algorithm for information transmission

**Fig. 2:**
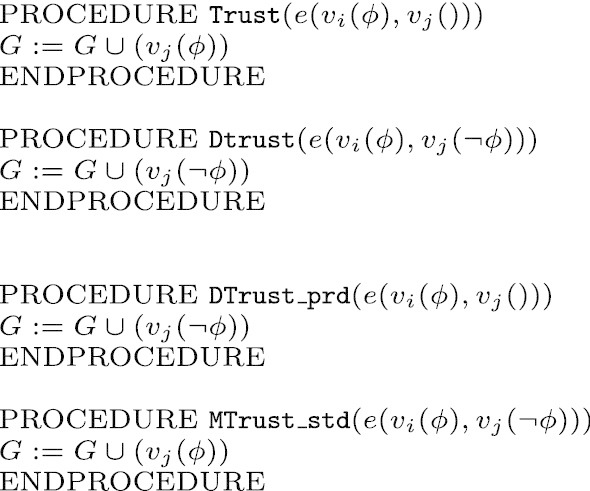
Algorithms for trust, distrust and mistrust

The formal model is then implemented in NetLogo, and the rules of the logic are initially applied to different graph’s topologies, and exchanges of information between the nodes correspond to formal derivations. A vertex $v_i$ in a graph *G* can be identified either as *standard*, *paranoid* or *sceptic*. The transmission between two nodes is expressed by an edge representing a *transmission channel*. A channel is denoted by $e(v_{i}(p),v_{j}())$; in this particular case, *i* transmits information *p* to *j*, where *j* represents a node not yet labelled (i.e. who holds no information). Different kinds of transmission are allowed, with distinct procedures to establish whether the information is accepted or rejected, depending on the epistemic attitude of the receiving agent. Differently from the logic, only atomic formulae *p* and $\lnot p$ are considered in the simulation; we establish by convention that *p* is the ground truth, while $\lnot p$ is a conspiracy theory. Every information-exchange between the nodes of the graph starts with a randomly seeded information *p*, which spreads across the network. The transmission of the information mimics the one of the logic, i.e. different operations are called by the standard and paranoid’s behaviors depending on their position in the hierarchy (see pseudocode in the Figs. 1, 2):

- a standard node *j* is labelled by *p* if linked to a node *i* holding *p* and *p* is consistent with *j*’s current information, i.e *j* holds *p* or *j* is not labeled yet; this procedure is executed by calling the $\mathtt {Trust}$ routine;
- a standard node *j* will accept *p* even if *p* is not consistent with *j*’s information, i.e. if *j* holds $\lnot p$, but is lower in the hierarchy than the node *i* sending the information; this procedure is executed by calling the $\mathtt {MTrust\_std}$ routine;
- if *j* is paranoid and receives information *p* from $i<j$, i.e. from higher in the hierarchy, *j* calls the $\mathtt {Dtrust\_prd}$ routine and the node is added to the graph with an opposite label $\lnot p$;
- when both paranoid and standard attitudes are simulated by vertices up in the ranking, information will be trusted if consistent, i.e it will be accepted, and will be distrusted if it is not consistent, i.e it will be rejected by calling the $\mathtt {Dtrust}$ routine;
- sceptic agents, when receiving $\lnot p$ from a paranoid agent, will reject it by applying the $\mathtt {Dtrust}$ routine.

For the purposes of the present study, the model for the misinfodemic is implemented in a scale-free network with a fixed number of nodes (500) through a simple linear preferential attachment algorithm in the Barabási-Albert style, with the hierarchy on vertices defined on the basis of their out-degree. This allows to identify the authority as the source of information with the largest connection and at the top of the hierarchy; but it also does not exclude that claques might be formed with paranoid agents filtering the information from the authority and hence having nodes who are entirely misinformed. In particular, a fixed distribution of the agents is considered in this study, as follows:

- 12% of paranoid agents;^1^
- 12% of sceptic agents, to balance the amount of paranoid agents;
- 76% of standard agents.

The simulation starts by seeding *p* in the highest ranked agent in the graph (the ground truth, or else policies dictated by the authority) and it starts spreading across the network. Following the procedural semantics illustrated above, upon receiving *p* paranoid agents will start to communicate the false information $\lnot p$.

## Simulation of a pandemic with misinformation

After having simulated the diffusion of misinformation, in order to identify paranoid agents as well as agents misinformed by paranoids, we then simulate an epidemic to investigate the effects of the former on the latter. Agents labelled by $\lnot p$ and therefore identified as paranoids or misinformed agents will constitute the part of the population which will refuse to comply with containment policies imposed by the government, thereby affecting the spread of the epidemic. The pandemic is modelled on real data referring to Italian population, including an average on its mortality rate for age groups and a simulation of containment strategies that build on the total lockdown imposed in Italy in period March–June 2020. For this simulation we allow randomly moving agents (i.e. without a graph structure), in order to implement both disease spread by proximity, and restrictions imposed by the authority.

The agents are divided in three groups defined by age, representing the demographic of the Italian population (http://dati.istat.it/):

- 18% of students: this group includes all agents who are in scholar age in an extended sense, i.e. between 0 and 20 years old; hence this value includes toddlers in daycare, while it does not account for children who are not scholarly active;
- 53% of workers: this group includes all agents who are potentially active on the labour market, i.e. between 20 and 60 years old; this value clearly does not account for unemployment rate;
- 29% of retired people: this group includes all agents who are aged 60 and above; this value only slightly extends the age range for retirement which begins in Italy at 67, but it allows to include the wide number of workers categories who benefit from pre-retirement agreements.

Agents groups defined by age are used to determine their movements, in terms of circles of different radius establishing their social interactions. Being within an agent’s perimeter indicates the occurrence of an interaction and a bigger radius means a higher daily average social interaction rate. In particular, we consider official data by the Italian Government Technical-Scientific Committee and assign an average daily contact rate (*dc*) as follows, see Scientifico (2020):

- $$dc(student)=4.5$$;
- $$dc(worker)=4$$;
- $$dc(retired)=2.4$$.

Additionally, we exploit the mean of their distinct daily contacts also to rule the passing of the days; a new day begins once each type of agent reaches the mean of their daily contacts reported above. These values are reached in average after 20 repetitions of the routine that regulates the movements of the agents.

We use the average of the infection period (*ip*) of 14 days for the COVID-19 virus as a standard for the length of the infection, see You et al. (2020). Agents are infected when in proximity. The probability for an healthy agent *i* of getting sick ($p(s)_{i}$) when sick agents are within her radius takes into consideration an $R_0=3.10$, reflecting the upper bound of the rate for COVID-19 calculated in the early phase of the outbreak in Italy, see D’Arienzo and Coniglio (2020). The $R_0$ value has obviously a different impact for each demographics, as it is parametrized by the average daily contacts given above. Hence

$$\begin{aligned} p(s)_{i}=\frac{R_{0}}{dc_{i}\times ip} \end{aligned}$$

We implemented an average of mortality rate (*mr*) by age on the Italian population due to COVID-19 reported in https://www.statista.com/statistics/1106372/coronavirus-death-rate-by-age-group-italy/:

- $$mr(student)=0.1\%$$;
- $$mr(worker)=1\%$$;
- $$mr(retired)=25.5\%$$.

The pandemic begins with just one agent sick and the virus spreads without control until $1\%$ of the population is infected. Once this threshold is reached, three containment measures may be applied:

- *Lockdown* Some agents are asked to not move;
- *Isolation* Sick people are detected and isolated;
- *PPE and Safety measures* Personal protective equipment is put in use, e.g. washing hands protocols, protective tools and distancing measures apply, which remarkably decrease the raw infectious rate of the virus.

We considered and implemented different possible lockdown options:

- *retired* retired people are asked to not move; the rationale behind this strategy is to keep economic and learning activities as much open as possible;
- *student* students do not move; this strategy is implemented to investigate the effect of closing down intensively populated structures like schools and universities, as well as reducing the impact of public transport as conducive means of the spread of the disease;
- *retired+student* both students and retired people will not move; it combines effects of both strategies above;
- *essential activities* only $50\%$ of workers is allowed to move, representing around $22\%$ of the total of the population; this strategy combines with the *retired+student* strategy above, and it is close to the strategy applied in Italy between 09 March and 03 June 2020 to block the COVID-19 pandemic;
- *all* everyone is asked to remain stationary.

Note that the disease still spreads from infected agents to agents in lockdown, as we admit interactions in households and minimal movements for essential services (food deliveries, visits to pharmacies and hospitals, and so on).

Agents who are identified as paranoids, or are misinformed by paranoids, will refuse to comply with governmental containment measures and will eventually break the lockdown. Daily, a fixed percentage of them are asked to go out anyway; this design choice allows to account for random irresponsible behavior of misinformed agents. We also model a scenario in which hard deterrents for breaking the rules are applied: these mimic the imposition of fines or the application of penal prosecution to those who do not comply. While in a normal scenario without deterrents up to $50\%$ of misinformed agents will break the lockdown, in the scenario with deterrents the proportion goes down to $10\%$. Additionally, if strict security personal measures are imposed, misinformed agents will also have an infection rate doubled compared to agents who have accepted the ground truth: this is intended to model their refusal to comply with the required use of PPE and safety measures.

Associated with the types of lockdown, we calculated a parameter expressing the social damage of keeping agents stationary: a ‘negative impact value’ *NIV* is assigned to each specific demographic of agents; if the relevant breed has been stationary due to the type of lockdown imposed, this value will be added to the variable keeping score of the general negative impact. One simple (and simplistic) way to account for such negative impact value is to assign a value for each agent who is not moving, weighted by its status: this grants that every non-active agent represents a cost for society (and for herself), and this cost is higher for workers, lower for students, and lowest for elderly and retired people. Moreover, the number of infected people concur to such value, as they directly impact the number of stationary agents. Hence we compute the NIV as:

$$\begin{aligned} NIV=\mid infected\mid \times (w \times \mid stationary\mid ) \end{aligned}$$

where the weight $w=1$ for workers, $w=0.75$ for students and $w=0.5$ for retired. Note that here we are discounting entirely the emotional costs of the lockdown strategies.^2^

While extended lockdowns should be discouraged, they represent a more desirable option than a quick but stronger epidemics causing the breakdown of hospitals. A malfunctioning health system will not only further increase the high mortality rate dictated by the virus, but it will also be unable to fulfill its ordinary duties. Thus, we assume that a long epidemics which remains sustainable for the health system will cause less damage than a fast but deadly one.

We halt the simulation when the infection has disappeared.

## Results

The experiments on the NetLogo simulation have been executed on a machine with 7.8 GB of memory running 64bit Windows 10. We run 50 repetitions of the simulation for each setting. To analyze the impact of the misinformation diffusion on the epidemic, we also run 50 repetitions for each setting without diffusion of misinformation. Hereafter we expose the main results.

### Security measures

The presence of security measures like PPEs, social distancing and detect and isolate policies are proven to be crucial to constraint the pandemic. Detection and isolation procedures simulate extensive trials on the population aiming at detecting sick people and put them on quarantine. This strategy has been applied in many European and Asian countries during the COVID-19 pandemic, in order to help constraining its diffusion.

**Table 1:** Control, no security measures, no detection

Lockdown	Infected	Deaths	Days
Retired	500	39	36
Student	500	40	35
Ret-stud	499	40	38
Essential	496	39	45
All	118	10	109
None	500	40	35

**Table 2:** Misinfodemic, no security measures, no detection

Lockdown	Infected	Deaths	Days
Retired	500	41	37
Student	500	40	36
Ret-stud	500	41	38
Essential	498	41	43
All	483	38	62
None	500	41	36

Our first study was aimed at showing the effects of lifting security measures of this type during a lockdown, see Table 1. In a scenario where no security measure nor detection policies are in place, the infection rate is almost always $100\%$, except with a total lockdown, corresponding to the all strategy in the Table, where it goes down to $24\%$ of the population. The duration of the pandemic is between 36 and 109 days in a total lockdown, in the latter case due to the fact that the infection is very constrained, while it keeps lingering on. In other words, if protective measures are not applied, all the other efforts produce no positive effects in controlling or limiting the diffusion of the disease. This result highlights the importance of protective measures during an epidemic: if they are not adopted, the benefits of keeping a fraction of the population stationary are eclipsed by the agents going out without protections.

In this scenario, we analysed the effect of the spread of a misinfodemic, see Table 2. This has an enormous effect precisely on the only type of lockdown in which the absence of protective measures has a limited impact, namely in a total lockdown. In this case, the role of paranoid agents is crucial and it manages to bring the infection rate up again to over $96\%$ and the mortality rate to values similar to those of the other types of lockdown. In this case, the only agents moving are those breaking the quarantine, thus they are the only vehicle of the disease. Hence, while the control model presents a significant reduction of infection and mortality rates with a total lockdown, these positive effects are neutralised in the model where misinformation occurs, due evidently to the impact that paranoids have by breaking the lockdown and misinforming standard agents. In this configuration, moreover, the length of the pandemic is much shorter when compared to the situation under total lockdown without misinformation, making the effects on e.g. healthcare infrastructure much heavier.

For comparison, we provide data of the same models when detection and isolation policies are applied (but no security measures like PPE). In the control model without misinformation, see Table 3: the infection and mortality rates are still very high under all types of partial lockdown, while they drop immediately under total lockdown, where only $6\%$ of infection rate and no fatalities occur. Adding a misinfodemics to this latter configuration makes those levels spike again, see Table 4: the mortality rate grows up to $55\%$, with 22 fatalities, i.e. $+\,20$ units over the previous scenario. This shows that the combination of a misinfodemic with a scenario in which policies related to protective measure and/or detection and isolation are missing, increases significantly the negative effects of the pandemic.

**Table 3:** Control, with detection and without security measures

Lockdown	Infected	Deaths	Days
Retired	478	37	37
Student	479	37	36
Ret-stud	468	37	37
Essential	425	33	43
All	31	2	31
None	481	37	35

**Table 4:** Misinfodemic, with detection and without security measures

Lockdown	Infected	Deaths	Days
Retired	480	37	37
Student	480	38	37
Ret-stud	464	36	38
Essential	446	34	42
All	275	22	70
None	480	39	37

### Lockdown strategies

Once individual protective measures are applied, the control setting shows remarkable differences compared to the simulation which takes also into account the diffusion of misinformation. In general, the number of infected agents is lower in all the settings for control. The graphs in this Section show the curves of the daily total infected agents.

**Fig. 3:**
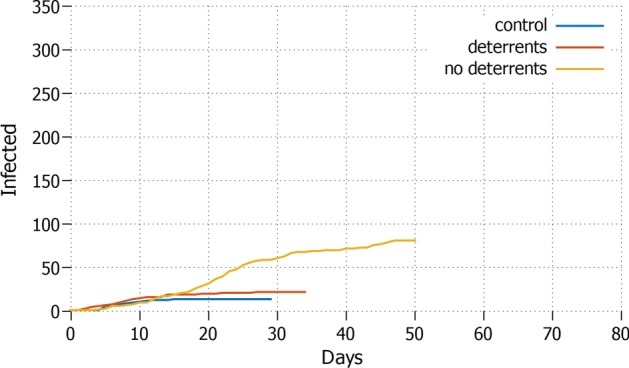
*All* Total daily cases with all agents in lockdown and protective measures applied

**Fig. 4:**
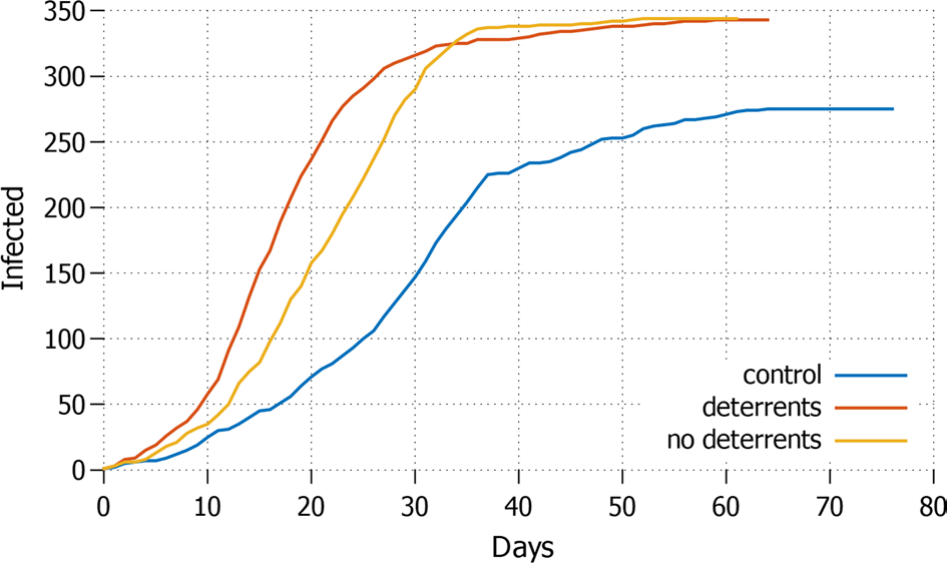
*None* Daily increment with no agent in lockdown and protective measures applied

We first compare the *all* and *none* strategies, i.e. respectively presenting the case where everyone is in lockdown, and no one is in lockdown. The strategy *all*—see Fig. 3—performs the best in terms of total infected and the duration of the epidemic: the infection rate remains well below the $1\%$ and the length of the pandemic remains within 35 days for both control (no misinformation) and the setting that has applied deterrents in the presence of misinformation. Hence, applying deterrents or hard deterrents has an important effect. As soon as no deterrents are imposed to counterbalance misinformation, major differences occur: the infection rate grows up to around $20\%$ of the population and the pandemic lasts almost twice as long.

Obviously, keeping all the agents stationary may not be a sustainable option. The opposite strategy *none*, where everyone is free to move, performs the worst, see Fig. 4. It shows the highest amount of infected agents, reaching almost $70\%$ of the population in around 50 days, which may cause an overcrowding of hospitals. In this scenario, the application of deterrents has no visible impact on how quick the pandemics evolves, and on its overall size. For comparison: the control setting without misinformation does not perform better on the duration of the pandemics, but the curve flattens earlier, keeping the size of the infection around $50\%$ of the population. This means that even in the worst possible condition of total absence of lockdown, guarding against misinformation diffusion can significantly help reducing the impact of the pandemic.

**Fig. 5:**
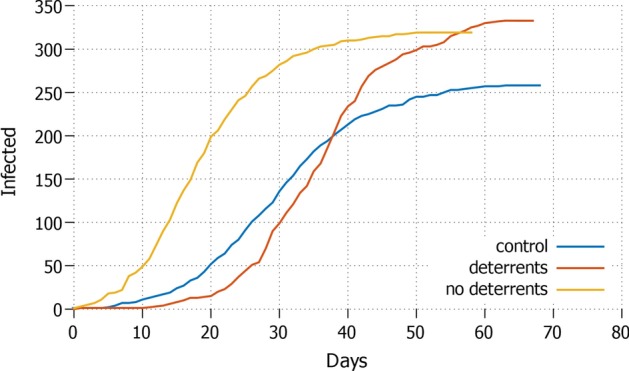
*Student* Daily increment with students in lockdown and protective measures applied

**Fig. 6:**
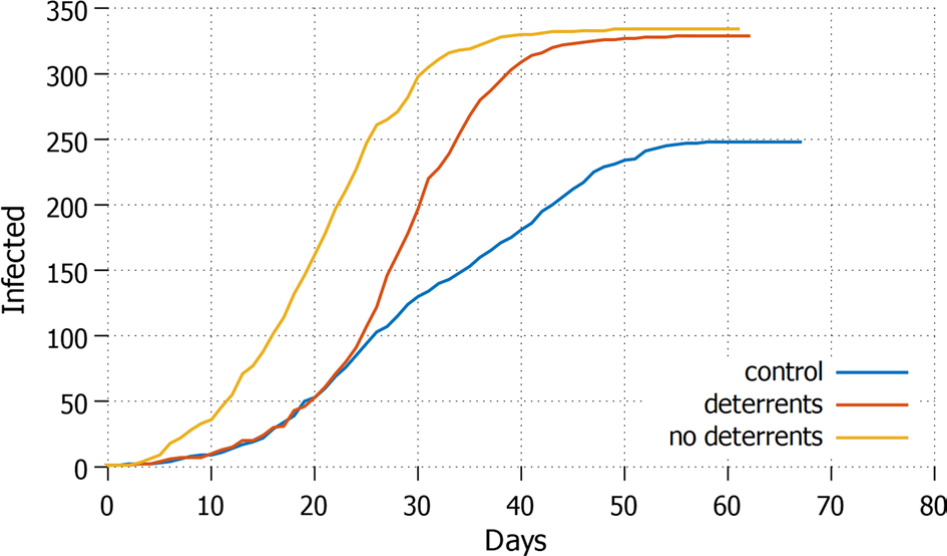
*Retired* Daily increment with retired people in lockdown and protective measures applied

The strategies *student* and *retired*, where respectively students and elderly and retired people are asked to remain in lockdown, are not especially effective in blocking the diffusion of the virus, see Figs. 5 and 6. In the former, the number of infected goes up to $65\%$ lasting for over 60 days in the control setting; in the latter this proportion goes up almost to $70\%$. Also the introduction of deterrents has only a limited effect on the size of the pandemic in the two scenarios. But pursuing a strategy where students are in lockdown (i.e. the demographic with higher contacts rate remains stationary), the curve of the infection rate grows slower than by keeping the elderly in lockdown. In both cases, the effect of misinformation control is relevant: in the control setting, despite a slightly longer pandemic (of about 20 days in the worst case), the number of infected agents is of about $15\%$ less when students are in lockdown, and of about $20\%$ less in the case of the *retired* strategy. This means that in both cases controlling misinformation diffusion has a positive effect, while the difference between the two is to be attributed to the demographic which remains stationary.

**Fig. 7:**
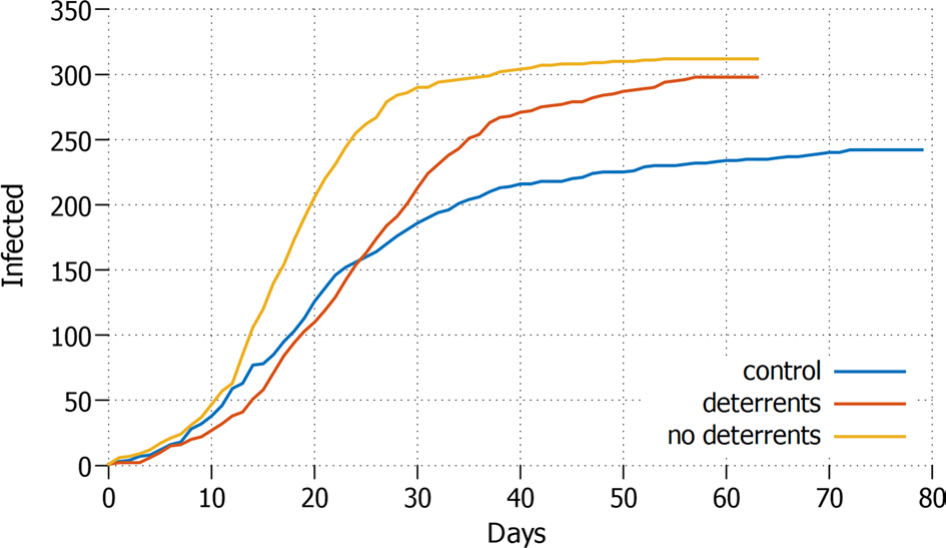
*Retired+student* Daily increment with elderly and student in lockdown and protective measures applied

**Fig. 8:**
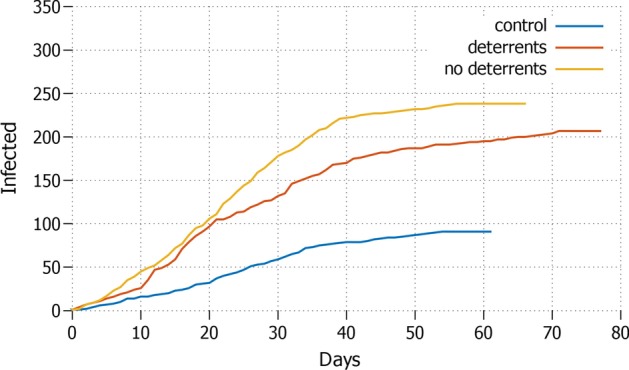
*Essential activities* Daily increment with only essential workers allowed to move and protective measures applied

In Fig. 7 we analyse a lockdown situation in which both retired people and students are asked not to move. While in the absence of misinformation this scenario presents a significant reduction in size of the pandemic of about 100 agents compared to the previous two strategies; this advantage is entirely lost in the presence of misinformation, where no significant positive difference is shown by the combined lockdown. Again, the presence of deterrents only helps slowing down the curve rising in the first 15 days.

The best outcome is observed when also a percentage of workers is kept motionless, mimicking the condition where only essential productive activities are maintained, see Fig. 8. In this case, the almost complete lockdown shows important advantages, bringing the pandemic size down in all scenarios. In particular, the difference with the control setting is staggering: without misinformation and in almost full lockdown the size of the pandemic grows only up to around $15\%$ of the population and is over within less than 70 days; in the worst case scenario of this configuration (essential activities only, misinformation and no deterrents) the difference can reach up to $+\,92,8\%$ in size and almost 20 days more in length.

It is interesting to note that the number of misinformed agents at the end of the pandemic does not strongly correlate with the number of infected agents: considering runs where individual protective measures are applied, the correlation between those two parameters remains low (0.17). This suggests that a low number of misinformed agents is sufficient to result in the huge negative impact highlighted above. This aspect is further analysed below in “Proportion of paranoid nodes” section.

### No detection and no isolation

In this section we explore the results of misinformation spread in a context where detection and isolation procedures are not applied during a pandemic. In this scenario, an agent who is not subject to the current lockdown policy, and who is becoming infected, cannot be identified and therefore will help spreading the disease.

**Table 5:** Control, no detection and no isolation

Lockdown	Infected	Deaths	Days
Retired	483	38	62
Student	483	40	62
Ret-stud	477	38	67
Essential	451	35	79
All	67	6	104
None	484	39	64

**Table 6:** Misinfodemic, no detection and no isolation

Lockdown	Infected	Deaths	Days
Retired	490	39	57
Student	491	39	57
Ret-stud	488	37	59
Essential	479	38	63
All	449	36	85
None	492	36	56

We use as benchmark the no detection and no isolation policy in the control setting, see Table 5: when compared with the control setting without security measures, the infection and death rates are very similar, namely around $95\%$ and between 7 and 8% respectively, execept for the total lockdown strategy *all*: here the values drop to $13\%$ and $1.2\%$ respectively. The length of the epidemic is between 62 and 104 days. Let us now consider the model with misinformation, see Table 6: again, there are no significant differences in terms of the number of deaths or number of infected, except for the case of the total lockdown strategy, where the presence of misinformation brings again the infection and mortality rates high up: $89\%$ and $7.2\%$ respectively. Moreover, in this case, the epidemic lasts less, i.e. the higher numbers are reached in a shorter period of time: this suggests that misinformation has a huge impact, also determining a much lower probability of stopping the epidemic if any detection and isolation would be put in place at any later point.

**Fig. 9:**
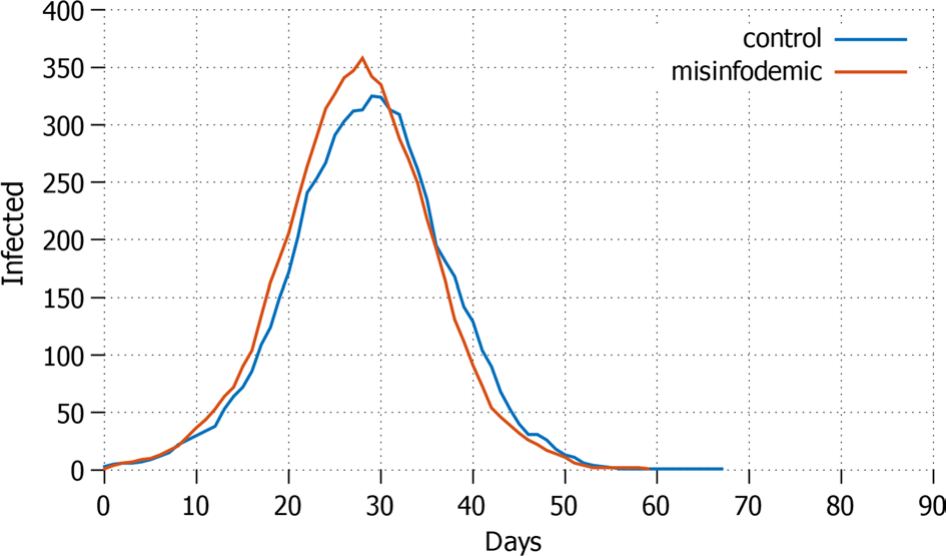
*Retired+student, protective measures applied without detection* Total cases without detection of infected with with retired and student in lockdown

In Fig. 9 we consider the *retired+student* strategy and no detection, i.e. a simulation of the situation in which those two categories of agents are under lockdown while any infected worker is left free to roam and thus helps spreading the pandemic. In this model, when misinformation is allowed the pandemic peaks to $75\%$ of the population in the space of nearly 30 days. On the other hand, in the control setting where no misinformation spread is in place, the peak is slightly less at above $60\%$ and it requires more than 30 days. Moreover, without misinformation the growth is slower.

**Fig. 10:**
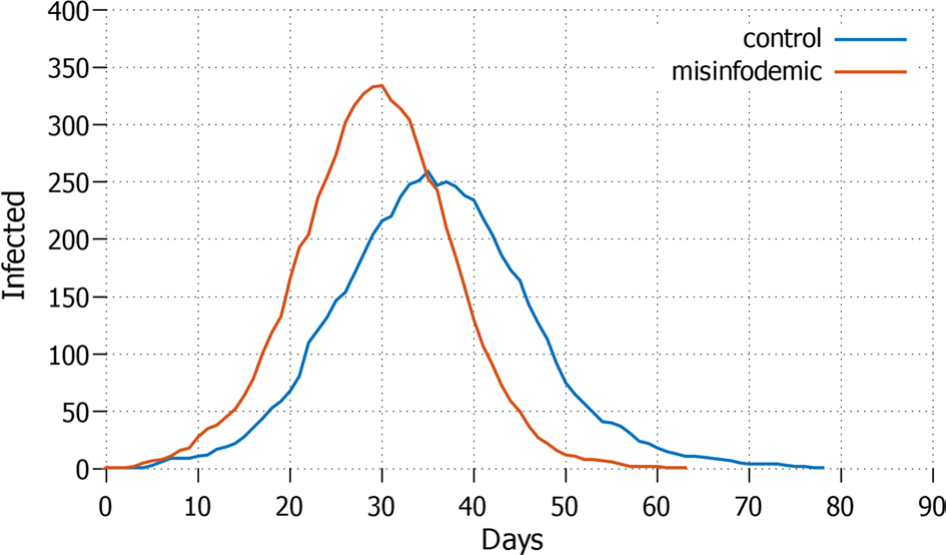
*Essential activities, protective measures applied without detection* Total cases without detection of infected and only essential workers allowed to move

In Fig. 10 we consider the *essential activities* strategy and no detection, i.e. where only around $22\%$ of the population is allowed to move and no control is performed on them, while everyone else is in lockdown. In this case, the difference between control setting and model with misinformation is in the range of $10\%$ less infected in the former, and the disease reaching its peak 15 days earlier in the latter. Again, the pandemic is faster in the presence of misinformation.

### Negative Impact

**Fig. 11:**
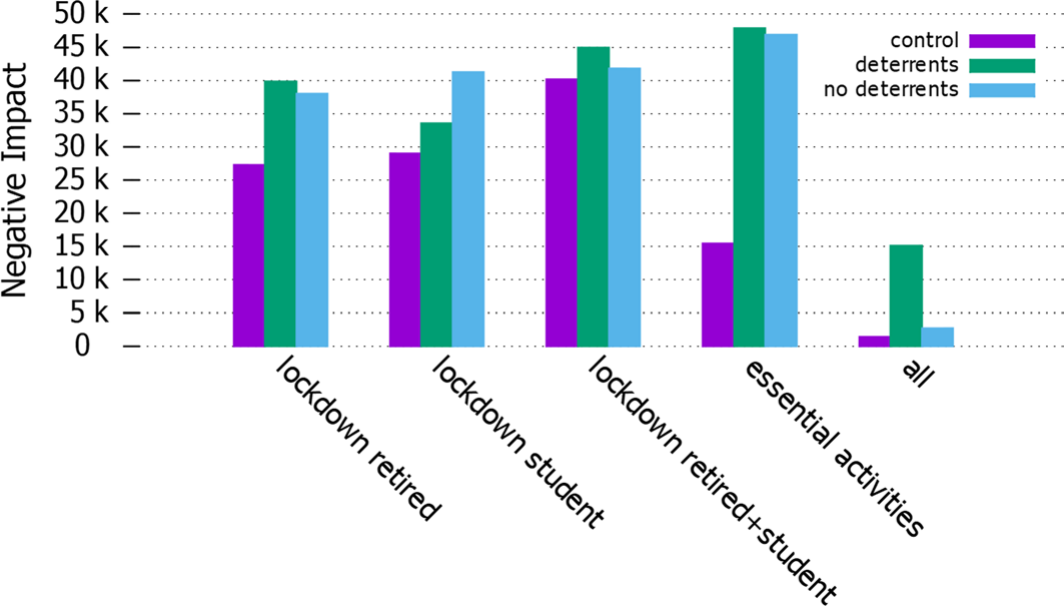
*Negative impact* Comparison of negative impact value in different settings

The pandemic, combined with the different types of lockdown, will produce a different negative impact at social and economic levels. In Fig. 11, we compare these values between the control setting and the model with misinformation (with and without deterrents).

**Fig. 12:**
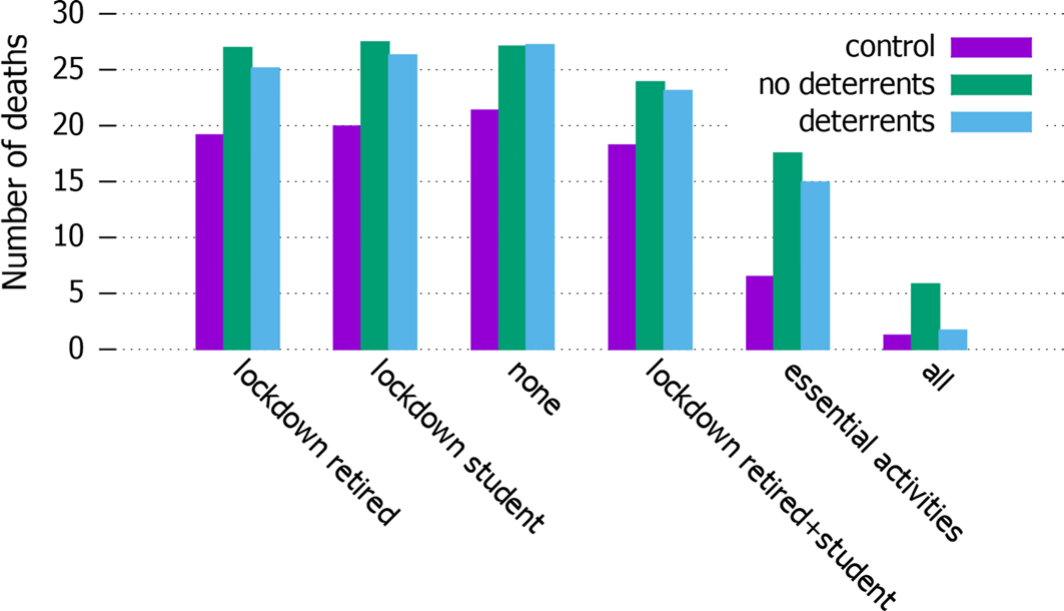
*Deaths* Number of deaths in the different settings

As exepcted, the lockdown type *all* shows the lowest negative impact, due to the low infection rate: it clearly indicates the benefit of keeping people stationary, despite the fact that it cannot be considered a sustainable strategy for a long time in a real-life situation, and also due to the fact that our NIV does not account for emotional distress. The NIV in the control setting is here the lowest in absolute terms, very close to the total lockdown with misinformation balanced by deterrents; where misinformation is present and no deterrents are enforced, the NIV grows rapidly. The *retired* and *student* lockdown strategies show similar values, with only a small increase due to the presence of agents breaking the rules due to misinformation spread. Similar considerations apply also for *retired+student* lockdown strategy, which however shows a general higher negative impact. In the control setting, the lockdown type which performs the best—besides the full lockdown—is *essential_activities*. However, the negative impact increases significantly when the diffusion of misinformation is taken into account in this setting. Thus, *essential_activities* becomes the most expensive strategy, clearly showing the damage of misinformation in a society during an epidemic, especially if not balanced by the imposition of deterrents.

We also compared the number of deaths for each setting and type of quarantine. The results are showed in Fig. 12. The control setting shows less deaths in all the quarantine types; the setting of no lockdown without deterrents to block people going out shows the highest number of deaths; the various partial lockdown strategies reduce this values, which goes down significantly in the total lockdown; but the presence of misinformation always contributes to the number of fatalities. The deterrent vs no-deterrent parameter seems to make a small difference in all settings but the one with total lockdown. First, considering that the parameter concerns deaths we assume even a small difference is significant. Moreover, we are studying only a small population, while with a much bigger sample the difference between the two will account for thousands of lives saved by the application of deterrents. In general, the lockdown options that show less difference between deterrent and no-deterrent options are the ones in which a large proportion of the population is still allowed to move. In these settings, the number of misinformed agents has actually a significant effect on the epidemics: as they are allowed to move as well as the informed ones, even if a proportion of the population applies personal safety measures they manage to have a significant influence. The case in which deterrence makes things even a little worse is where everyone is allowed to move. We ascribe the small difference between the two settings to a grater diffusion of misinformation, which is also linked to a random factor of communication among nodes: in particular, a greater diffusion of misinformation wil obviously occur when misinformed nodes are enough central in the network to influence a large number of agents.

### Proportion of paranoid nodes

**Fig. 13:**
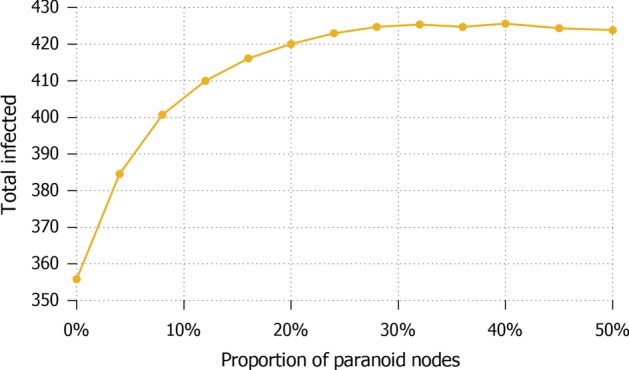
*Proportion of paranoid nodes (a)* Different paranoid nodes distribution compared with the number of total infected

**Fig. 14:**
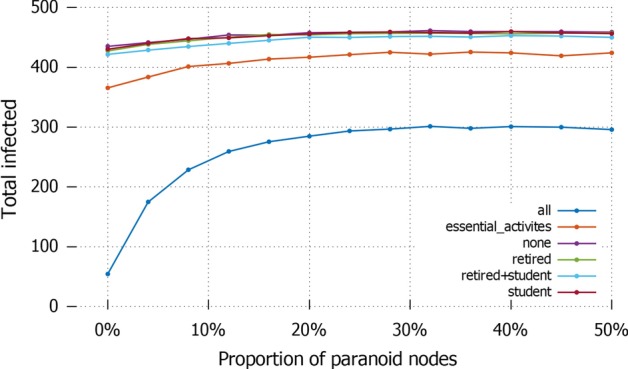
*Proportion of paranoid nodes (b)* Different paranoid nodes distribution compared with the number of total infected by lockdown type

The analysis developed so far accounts for a static analysis of the population’s attitude towards information. In other words, the number of paranoid agents remains fixed over time, and only the attitude towards information of agents change, primarily of standard agents who get influenced by paranoid ones. This obviously has the limitation of not considering possible variations in the fraction of population which is considered the cause of misinformation spread. In fact, this number can be different across different cultures and countries, and also the situation they experience and the results obtained by specific strategies can have an effect on how people decide to start trusting (or stop trusting) the information shared by the authority. While the logic underlying our model does not formalize such change of mind, we have studied the effects of the number of paranoid nodes in the number of total infected, to consider a first direct correlation between the two parameters. In Fig. 13 these two parameters are simply compared as an average on all the various settings proposed in this study: here we see that there is a linear increase of infected in the number of paranoid agents, but there exists a tipping point between 25 and 30 units after which a higher number of paranoid agents no longer has an effect; this seems to suggest that the worst effect of misinformation diffusion is reached already with a population of paranoid agents amounting to at most $6\%$. In Fig. 14 the same comparison is analytically offered under the different lockdown strategies. Here we see an even lower bound to the correlation: for the total lockdown the number of paranoid becomes irrelevant after 25 units ($5\%$ of the population), while in limited lockdown settings this number goes down to roughly 10 units ($2\%$ of the population).

## Conclusions

We studied the role of misinformation diffusion in helping spreading a pandemic. In general, misinformation increases the rate of infected agents in the population in nearly all the models presented. Specifically, if measures are applied in order to block the epidemic, the presence of misinformation has a negative impact on their effectiveness. The results highlight the importance of personal protective measures, lockdown, and detection and isolation policies also in compensating the negative effects due to misinformation spread, and in turn disease diffusion. When protection, distancing measures and detection and isolation policies are not applied, misinformation increases infection and death rates even in the presence of a full lockdown. The partial lockdown type which performs the best is the strategy which keeps the three demographic stationary, allowing only $50\%$ of workers to move. The other lockdown types show marginal effects if they are compared to the efforts of applying them. Applying hard deterrents to prevent agents from breaking the lockdown does not have major positive effects in controlling the epidemic in the presence of misinformed agents. This suggests that the negative impact of misinformation is not due to a high initial number of paranoids, and that a few agents not respecting the rules are enough to cause major differences. In some cases, misinformation has the effect of speeding up the epidemic, which in a real-life situation may cause an overcrowding of hospitals, with difficulties in the hospitalization of the infected. The effects of misinformation are not limited only to the infection rate: the negative impact of the strategies applied in order to block the epidemic is higher in all the setting when paranoid agents are present in the system.

We foresee a number of extensions and improvement to this model. We have not taken in consideration the possibility of preventive measures against the diffusion of the virus. In the current setting, the containment measure are applied when $1\%$ of the total population is infected. Therefore, we evaluated the impact of misinformation in a situation of emergency. However, it would be also compelling to study the effects of misinformation in a setting more oriented on preventive measures to block a possible outbreak of the virus. Moreover, our agents are epistemically stubborn, and their attitude does not change in the presence of new information, especially related to the effectiveness of containment measures: it would be a significant improvement the extension of this model with a counteracting information activity which might induce previously misinformed agents to change their attitude. This would allow to trace back the curves illustrated in Figs. 13 and 14. Finally, on a conceptually harder issue, it would be interesting to identify conditions under which paranoid agents can have a healthy effect, in particular when the authorities are not trustworthy or subject to mistakes due to inaccuracy, incompetence, or incomplete and misinterpreted information.

## Data Availability

The datasets generated and/or analysed during the current study are available in the GIT repository https://github.com/LorenzoPrandi/Paranoid

## Abbreviations

*dc*: Daily contact rate
*ip*: Infection period
*mr*: Mortality rate
*NIV*: Negative impact value
*PPE*: Personal protective equipment
$p(s)_{i}$: Probability for an healthy agent *i* of getting sick
*prd*: Paranoid agent
*std*: Standard agent
$R_0$: Basic reproduction number

## Footnotes

1 While the core data for the experimental analysis of this study is based on the Italian population, for this particular but crucial aspect statistical data is not precisely matching: in a study from 2015, the category of people older than 15 years of age suffering from depression accounts for $4.3\%$, while $1.6\%$ suffers from major depressions, see https://www.istat.it/it/files/2015/10/Salute-mentale_Giorgio-Alleva_2017.pdf. Unfortunately this category is much coarser than what we identify as a paranoid personality, which does not necessarily imply further mental health issues. The data we use represents people in the population of the U.S. who endorse at least three conspiracy theories, see Oliver and Woorod (2014).

2 Besides discounting for emotional costs, this index cannot offer a nuanced analysis of the trade-offs between different costs (e.g. risks to lives by infection vs. risks to lives by poverty as a second-order effect of the same cause). With this first approximation, we focus only on a combination of values for costs due to infection and costs due to stationary agents, the latter intended as a proxy for economic costs.
